# From Intention to Execution: Pre‐Race Nutrition Behaviours, Influences, and Performance Outcomes in Female Endurance Athletes at the IRONMAN World Championships

**DOI:** 10.1002/ejsc.70193

**Published:** 2026-06-11

**Authors:** Harvey O. Fortis, Amy E. Whitehead, Colum J. Cronin, Kelsie O. Johnson, Sam O. Shepherd, Anthony C. Hackney, Graeme L. Close, Juliette A. Strauss

**Affiliations:** ^1^ Research Institute for Sport and Exercise Sciences (RISES) Liverpool John Moores University Liverpool UK; ^2^ Precision Fuel and Hydration Ltd. Christchurch UK; ^3^ Department of Exercise and Sport Science University of North Carolina Chapel Hill North Carolina USA; ^4^ Department of Nutrition University of North Carolina Chapel Hill North Carolina USA

**Keywords:** carbohydrate loading, exercise performance, female endurance athlete, hydration, nutrition behaviours, physiology, ultra endurance

## Abstract

Endurance competition preparation involves complex psychological, logistical, and sociocultural factors. This study investigated the pre‐race nutrition practices of female endurance athletes, by applying a newly developed extended Theory of Planned Behaviour, including behavioural execution to the framework to capture real‐world influences (ETPB‐X). Using a convergent mixed‐methods design, 27 female triathletes competing at the 2024 IRONMAN World Championships completed questionnaires, 48‐h food diaries, and semi‐structured interviews before and after the race. Quantitative data (*n* = 23) were analysed for energy and macronutrient intake, whereas qualitative data were thematically coded using the conceptual ETPB‐X framework, incorporating attitudes, subjective norms, perceived behavioural control (PBC), utilitarian drivers, and behavioural execution. Only 26% (6/23) of athletes achieved carbohydrate loading guidelines (8–12 g·kg^−1^ body mass (BM)·day^−1^), with an overall mean intake of 6.4 ± 2.1 g·kg^−1^ BM. Higher carbohydrate intake correlated with faster finish times (*r* = −0.50, *p* = 0.035), whereas fibre intake was positively associated with gastrointestinal (GI) symptom severity (*r* = 0.85, *p* < 0.001). Qualitative findings revealed that adherence to diet plans was influenced by PBC, travel logistics, emotional regulation, and athlete identity. This study provides a novel application of the ETPB‐X framework to pre‐race nutrition in female endurance athletes. Athletes often under‐achieve carbohydrate loading targets despite awareness, with success determined as much by psychosocial and contextual factors as by knowledge. Practically, enhancing PBC, improving planning/preparation, and delivering clear goal‐aligned education may bridge the intention‐behaviour gap. Integrating behavioural frameworks into performance nutrition offers a pathway towards more effective athlete‐centred interventions.

## Highlights

- Pre‐race carbohydrate loading was frequently suboptimal in female endurance athletes, with only 26% meeting guideline intakes, despite widespread awareness of recommendations, and higher intake was associated with faster race completion times.
- GI symptoms were common and associated with higher pre‐race fibre intake, highlighting a disconnect between public health dietary messaging and sport‐specific nutrition practices during competition preparation.
- Translation of nutrition intentions into action was strongly influenced by perceived behavioural control, logistical constraints, emotional regulation, and competition‐specific pressures rather than nutritional knowledge alone.
- Applying a novel, conceptual, Extended Theory of Planned Behaviour–Execution (ETPB‐X) framework revealed that athlete identity, reflection, and contextual factors are central to pre‐race nutrition behaviour in female endurance athletes.

## 1 Introduction

Acute preparation for endurance competition is a complex and multifaceted process. Athletes must manage training taper, psychological readiness, logistical arrangements such as travel and accommodation, and event‐specific demands, including registration and equipment preparation. Nutrition is a central part of this preparation but does not occur in isolation. Decisions about pre‐race fuelling are made within the context of these broader pressures, and are influenced by practical constraints, family and social obligations, and the cultural rituals surrounding endurance sport. Understanding nutrition in this holistic context is essential, since even well‐designed dietary strategies can be compromised by the realities of competition preparation.

Despite the well‐established benefits of carbohydrate loading for endurance performance (Hawley et al. 1997; Burke et al. 2011; Thomas et al. 2016), female‐specific research remains limited. A recent audit of acute carbohydrate fuelling studies reported that females comprise only around 11% of all participants in this audit, with approximately 4% conducted exclusively in female cohorts (Kuikman et al. 2023). Available evidence suggests that women possess a comparable glycogen‐storage capacity to men when carbohydrate intake is matched relative to BM (McLay et al. 2007). However, observational research repeatedly shows that female endurance athletes habitually under‐consume carbohydrate, potentially compromising glycogen storage and the potential effectiveness of loading strategies (Logue et al. 2018; Sampson et al. 2023, 2024). Consensus guidelines recommend 8–12 g·kg^−1^ BM of carbohydrate in the 24–48 h before competition (Sedlock 2008; Thomas et al. 2016). Despite wide‐spread awareness of these recommendations, many endurance athletes fail to meet them, typically consuming only ∼2.5–7 g·kg^−1^ BM during this period (Sampson et al. 2023; Heikura et al. 2017). Female‐specific data are particularly scarce, although existing evidence indicates that pre‐race carbohydrate intake commonly remains below ∼6.5 g·kg^−1^ BM (McLay et al. 2007). Together, these findings reveal a gap between physiological potential and real‐world practices, reinforcing the need to explore the behavioural, contextual, and psychosocial factors that influence the process of carbohydrate loading in female endurance athletes.

In parallel, GI distress is common during endurance events, affecting both performance and athlete experience (Costa et al. 2016, 2017; Pugh et al. 2018; Ribichini et al. 2023). Pre‐race nutrition can strongly influence symptom risk, with fibre reduction widely recommended to minimise discomfort (de Oliveira and Jeukendrup 2014). Mechanistically, higher fibre intake is proposed to increase gut residue and fermentation, elevate colonic gas production, and accelerate intestinal transit, collectively leading to a greater risk of bloating, cramping, and urge to defecate during exercise (de Oliveira and Jeukendrup 2014; Costa et al. 2017). However, the advice to reduce fibre intake in the pre‐race period, contrasts with general public health messaging, where high fibre intake is promoted as a marker of good diet quality (WHO/FAO 2003; EFSA Panel on Dietetic Products and Nutrition and Allergies (NDA) 2010). For athletes, particularly those without formal nutrition support or education, this distinction between nutrition for health and nutrition for performance may not always be clearly recognised. High‐fibre foods are often marketed as ‘healthy’ choices, making intentional reduction counterintuitive for some. Female athletes may face additional challenges in this respect, as they typically report higher rates of both GI symptoms and pre‐race anxiety compared with men (Wilson 2017; Wilson et al. 2023), which should be considered in nutritional recommendations.

These observations highlight the importance of evidence‐based guidelines for carbohydrate loading and fibre reduction to optimise physiological readiness prior to competition. However, pre‐race nutrition practices are shaped by more than physiological requirements alone. Athletes must interpret, prioritise, and implement these recommendations within the practical and psychological constraints of competition preparation. Decisions are therefore influenced not only by physiological output or knowledge of what is recommended but also by athletes' beliefs and background, their ability to implement strategies, social expectations, and the situational and contextual influences which surround race preparation. To systematically examine how these factors interact to shape pre‐race nutrition practices, a theoretically informed framework is required.

### 1.1 Theoretical Framework

The Theory of Planned Behaviour (TPB) (Ajzen 1991) is a widely applied and well supported framework for understanding human decision making. It proposes that behaviour is driven by three primary constructs: attitudes, which are individual's beliefs about the outcomes of a behaviour and its perceived value; subjective norms, which are the social pressures and expectations influencing behaviour; and perceived behavioural control (PBC), which are the perceived ease or difficulty of performing the behaviour, reflecting both internal capability and external constraints. Together, these variables shape behavioural intention, which in turn leads to whether the intended behaviour is performed, or not. The TPB is valued for its ability to link psychological influences with actions, making it well suited to exploring athlete decision‐making and behaviour in complex and multifactorial real‐world environments.

**FIGURE 1:**
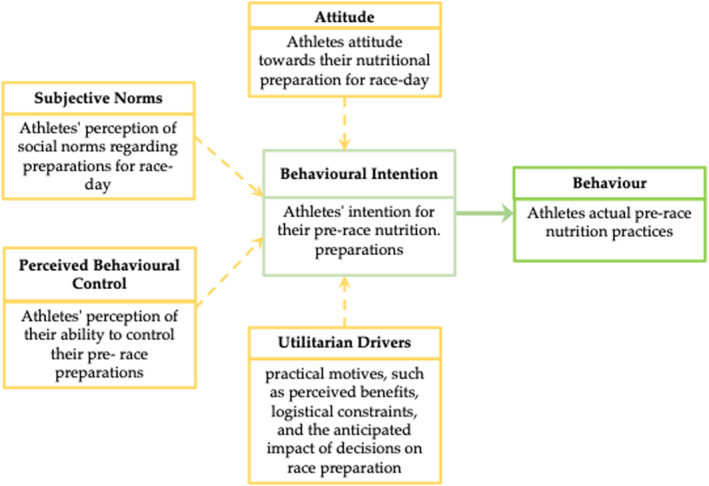
The Extended Theory of Planned Behaviour model adapted for pre‐race nutrition. Adapted from Samoggia and Rezzaghi (2021), this framework illustrates how attitudes, subjective norms, perceived behavioural control, and utilitarian drivers (yellow) contribute to athletes' behavioural intentions (light green), which in turn influence pre‐race nutrition behaviours (green). This adapted version incorporates the context of endurance competition preparations.

To address the limited psychosocial understanding of pre‐race nutrition, this study applied an extended version of TPB (ETPB), as created by Samoggia and Rezzaghi (2021) (Figure 1). Their model introduces ‘utilitarian drivers’, as an important influencing construct to behaviour outcomes. Utilitarian drivers are practical and comfort‐based influences on decision‐making that account for everyday realities of athletes' nutrition choices. Building on this framework, we developed the Extended Theory of Planned Behaviour‐Execution Framework (ETPB‐X). The ETPB‐X is a conceptual model, which introduces the additional variable of ‘behavioural execution’, recognising that the translation of intention into action is shaped by situational, logistical, and psychological influences during competition preparation. This adaptation provides a more applied account of pre‐race nutrition behaviour, capturing not only what athletes plan to do but also how those plans are performed under real‐world pressures.

Despite a growing body of research in endurance nutrition, important gaps remain. Most studies on pre‐race preparation have focussed on macronutrient intake, with limited attention to the lived experiences and contextual challenges that shape behaviour. Female endurance athletes remain particularly underrepresented, despite their distinct physiological and psychosocial considerations (Logue et al. 2021). Furthermore, existing research typically employs either quantitative or qualitative designs, rarely integrating both. Mixed‐methods approaches are uniquely positioned to bridge this divide by linking what happens (dietary intake data) with why it happens (psychological and contextual insights). Accordingly, this study integrated dietary intake data with qualitative interviews through the ETPB framework, we aimed to explore not only carbohydrate loading, macronutrient, and fibre manipulation but also the broader contextual, psychological, and sociocultural factors influencing nutrition practices in the days preceding IRONMAN competition. This investigation represents the first phase of a two‐part series examining endurance nutrition across the competition period, with the subsequent study focussing on the execution of in‐race fuelling strategies and behavioural adaptations within the same athlete cohort.

## 2 Materials and Methods

### 2.1 Design

This study adopted a triangulation and a convergent mixed methods design, as outlined by Bishop (2015) and was underpinned by a pragmatic philosophical position, recognising the value of combining quantitative and qualitative data to address applied research questions. The convergent design involves simultaneous collection and analysis of quantitative and qualitative data streams, allowing for comparisons or relations to be made between data sets. Both data types were given equal priority, reflecting a commitment to a balanced mixed‐method approach. Quantitative and qualitative results were integrated during the interpretation phase to holistically explore female endurance athletes' nutritional preparation for endurance competition.

To gain detailed insights into the perceptions and practices of the population of interest, female endurance athletes were purposefully recruited on the basis that they were aged > 18 years and registered to compete at the 2024 IRONMAN World Championships. This approach is comparable to previous qualitative explorations of nutrition practices in sport (Martin et al. 2014; Logue et al. 2021; McHaffie et al. 2022; Fortis et al. 2025). Recruitment occurred through two routes: (1) direct contact via social media platforms and triathlon clubs and (2) convenience sampling through participant networks and race‐related contacts. All athletes received participant information before enrolment. In total, 27 athletes completed the initial questionnaire, 23 athletes completed the 48 h food diaries, and 27 athletes participated in the individual interview components of the study, with four athletes providing incomplete food diaries (e.g., missing meals, weights, poor image quality) and were therefore removed from this component of the study. All participants provided verbal and written informed consent before completing the food diaries and interviews. Consistent with qualitative research principles (Sparkes and Smith 2014), sample size was not predetermined but emerged through the analytic process. Recruitment continued until no new insights were generated and thematic saturation was reached. In line with contemporary guidance, we also drew on the concept of informational power (Malterud et al. 2016), which proposes that the more relevant, rich, and focussed the data are to the study aim, the fewer participants are required. Given the specificity of the cohort, the open dialogue from participants, and the depth of the interview material, informational power was deemed high, supporting the decision to halt recruitment once consistent patterns were established and additional interviews no longer contributed novel information.

Participants completed an initial questionnaire to provide personal information and to validate their eligibility to participate. Of the 27 individuals who responded to the initial questionnaire, 16 self‐reported to be peri‐ or post‐menopausal. Of the 11 premenopausal athletes, 36% (*n* = 4) reported using hormonal contraception (x2 = a variation of the pill and x2 = Intrauterine device), and 64% (*n* = 7) were not using hormonal contraception, and all reported having a normal menstrual cycle (21–35 days). Menopausal status and hormonal contraceptive use were recorded to characterise the cohort and acknowledge potential physiological heterogeneity; however, the study was not designed or powered to examine hormonal‐status‐specific differences in dietary behaviour or performance outcomes.

Participant characteristics were as follows: (mean ± SD): age, 45 ± 12 years; BM, 60.1 ± 3.9 kg; height, 155.9 ± 13.3 cm. Training volume (per week, in race preparation) of participants were as follows: 7–9 h, 7%; 10–12 h, 33%; 12–15 h, 22%; and > 15 h, 37% and ranged from 2 to 30 years of triathlon‐specific training history, with previous best IRONMAN completion times of 12:10:43 ± 1:27:18 (Table 1).

**TABLE 1:** Breakdown of age groups and training volume classification of participants.

	Number of participants (*n* = 27)
Age group (years)	
18–29	4
30–39	6
40–49	7
50–59	7
> 60	3
Training volume (h/week)	
6–9	2
9–12	9
12–15	6
> 15	10

### 2.2 Pre‐Race interviews

Semi‐structured interviews were conducted < 1 month prior to the race (interview time = 00:50:01 ± 00:10:50, hh:mm:ss) to explore participants' pre‐race nutrition plans, perspectives, and influences. The interviews aimed to identify the athletes' intentions, behaviours, and perceptions related to their nutrition strategies. The interview format followed an open‐ended approach, allowing for a conversational and flexible discussion (Gall et al. 2003). Examples of interview prompts included: ‘What are your thoughts on…?’ and ‘How would you plan to…?’ Probing questions (see supplementary material) were used to elicit further details, and the researcher maintained a non‐directive stance to encourage participants to express their own perspectives (I. Jones 2022).

The development of interview questions was guided by the ETPB model (Samoggia and Rezzaghi 2021), which expands upon the traditional TPB by incorporating additional psychosocial factors influencing decision‐making. Specifically, questions were designed to explore key components of the model, including attitudes, subjective norms, perceived behavioural control, and utilitarian drivers (Figure 1), specific to athletes' race preparations. This theoretical underpinning ensured a comprehensive exploration of the psychological and social factors shaping athletes' pre‐race nutrition behaviours.

### 2.3 Post‐Race Interviews

Follow‐up semi‐structured interviews were conducted within 10 days after the race (interview time = 00:34:18 ± 00:12:52, hh:mm:ss) to gather contextual insights into the athletes' actual nutrition behaviours and experiences. These post‐race interviews aimed to evaluate how athletes executed their pre‐race plans, the challenges they encountered, and the external or internal influences that shaped their nutrition strategies. Example questions include ‘How closely were you able to follow the nutrition plan you had intended before the race?’ and ‘Were there any unexpected challenges or changes that affected your nutrition on race day?’. Specific attention was given to identifying both positive and negative factors affecting decision‐making, such as environmental constraints, physiological responses, and psychological influences. All interviews were conducted via online video conferencing (Microsoft Teams, California, USA), and all sessions were audio‐recorded. Each interview took place in a private setting, ensuring confidentiality and creating a comfortable space for participants to share their insights. Data were transcribed verbatim, and transcripts were reviewed and refined to ensure accuracy.

As a part of an additional study, nutritional recall was obtained immediately after the race. This included subjective measures of GI symptoms during the race, with a focus on bike and run segments, using the GI Symptom Questionnaire (Wilson 2017). This validated tool captures six common symptoms: nausea, regurgitation/reflux, stomach fullness, abdominal cramps, gas/flatulence, and urge to defecate, rated on a 0–10 visual analogue scale. Scores of ≥ 4 were considered to represent symptom severity likely to impair exercise performance. Immediate post‐race data collection has been shown to capture valid and reliable assessments of GI symptoms in endurance athletes, supporting the retrospective accuracy of this method (Wilson 2017). This has been used in the present study as a means to assess trends in pre‐race nutrition practices' effects on GI symptoms.

### 2.4 Qualitative Data Analysis

**FIGURE 2:**
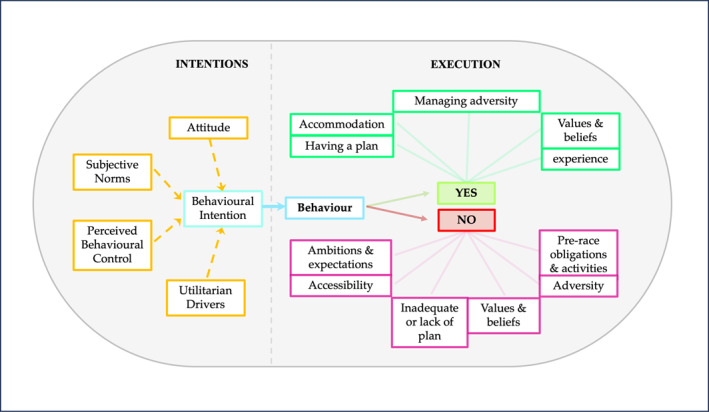
Extended Theory of Planned Behaviour–Execution Framework (ETPB‐X) illustrating determinants of pre‐race nutrition behaviour in endurance athletes. This adapted conceptual framework builds upon the Extended Theory of Planned Behaviour by incorporating behavioural execution to capture the translation of planned nutrition strategies into real‐world behaviour during race preparation. Traditional TPB constructs (attitudes, subjective norms, perceived behavioural control) together with utilitarian drivers (yellow boxes) influence behavioural intentions (light blue). Behavioural execution (blue) represents the implementation of intended nutrition behaviours within the practical constraints of competition preparation. Green elements represent factors that facilitated successful execution (e.g., logistical control, planning, prior experience), whereas pink elements represent barriers that impeded adherence (e.g., travel constraints, social obligations, anxiety, or food access). Arrows illustrate the directional relationships between constructs and behavioural outcomes.

The qualitative data were analysed using a deductive framework developed to assess the execution of planned behaviour (Figure 2), examining whether athletes adhered to their intended strategies and the determinants of successful or compromised execution. Following verbatim transcription, meaningful text segments were identified according to the interview domains, and initial open coding was conducted (Saldana 2021).

An initial codebook of 58 descriptive codes was generated and refined through iterative review and discussion. These codes were grouped into 18 subthemes and organised within six overarching themes corresponding to the constructs of the ETPB‐X: attitudes, subjective norms, perceived behavioural control, utilitarian drivers, behavioural intentions, and behavioural execution; the final framework was reviewed and agreed upon by all authors.

### 2.5 Rigour in Qualitative Analysis

To ensure the rigour and credibility of the qualitative findings, several strategies were employed. The lead researcher, who had training in qualitative research and experience as a nutritionist and endurance athlete, conducted the interviews. Interview questions were pre‐tested through pilot interviews and reviewed by a critical friend to ensure questions were clear and avoided leading participants' responses. The coding process and theme development were scrutinized by multiple critical friends, who provided diverse perspectives, including those with expertise in nutrition and qualitative research. This critical feedback ensured reflexivity and enhanced the validity of the analysis (Smith 2018). Examples of this process include the development of the behavioural execution component of the model. Members of the critical friend process also highlighted the importance of establishing athletes' previous experience as a key driver of behaviour outcomes. The research team consisted of a nutritionist, psychologist, and qualitative research expert, bringing a diverse skill set and expertise. This multidisciplinary perspective (physiological, sociological, and psychological) enabled more balanced and comprehensive interpretations of the data.

### 2.6 Quantitative Data Collection

Quantitative data were collected using a combination of weighed food diaries and the remote food photography method (RFPM), specifically designed to capture athletes' food and beverage intake during the 2 days leading up to the race and the race day morning (Martin et al. 2008). Athletes were instructed to weigh all food and drink consumed, and they submitted photographs of their meals along with descriptions, including details of food preparation and quantities. These data were submitted via WhatsApp (Dublin, Ireland) and analysed for energy and macronutrient intake.

### 2.7 Quantitative Data Analysis

Dietary intake was analysed using Nutritics software (Dublin, Ireland), which calculated energy and macronutrient intake. All dietary records were independently analysed by a trained nutritionist with over 5 years of experience. A secondary researcher checked 25% of the entries to ensure data accuracy. The results were averaged to provide daily estimates of energy intake (kcal/day) and macronutrient breakdown (g and g·kg^−1^BM).

### 2.8 Integration of Qualitative and Quantitative Data

The integration of qualitative and quantitative data followed a concurrent triangulation design (Creswell and Plano Clark 2018). After individual analysis of both data sets, the findings were compared to identify patterns, contradictions, and complementary insights between the athletes' reported behaviours and their actual dietary intake. This process helped to deepen the understanding of athletes' perspectives on their pre‐race nutrition and to identify any discrepancies between planned and actual nutrition behaviours.

### 2.9 Ethical Considerations

This study was conducted in accordance with ethical guidelines, and all participants provided informed consent prior to participation. Institutional ethical approval was granted by the ANON Research Ethics Committee, UREC reference: ANON. Participants were assured that their identities would remain confidential and that all data would be anonymized.

## 3 Results

### 3.1 Quantitative

Most athletes did not achieve the recommended carbohydrate intake of 8–12 g·kg^−1^ BM during the final 24–48 h before competition (Table 2). Only 6 of 23 participants (26.1%, 95% CI: 12%–46%) met this guideline. Reported carbohydrate intakes varied substantially, ranging from 2.8 to 9.9 g·kg^−1^ in the 24 h preceding the race. Mean carbohydrate intake increased from 3.9 ± 1.6 g·kg^−1^ on Day −2 (95% CI: 2.72–5.04) to 6.4 ± 2.1 g·kg^−1^ on Day −1 (95% CI: 5.51–7.23).

**TABLE 2:** Energy and macronutrient intake for the 48 h prior to competition (*n* = 23), separated into the 24 h, 24–48 h, and average over the 48 h. Where applicable, data are presented as total (*g*) and relative to BM (g·kg‐1 BM).

		Total					Relative		
		Energy (kcal)	CHO (g)	Fat (g)	Protein (g)	Fibre (g)	CHO (g·kg^−1^)	Fat (g·kg^−1^)	Protein (g·kg^−1^)
Day −1	Mean	2727.9	379.6	104.4	87.1	21.9	6.4	1.7	1.4
	SD	790.6	132.3	35.7	37.7	6.9	2.1	0.6	0.6
Day −2	Mean	2059.6	236.1	94.7	81.8	18.9	3.9	1.6	1.4
	SD	677.7	103.9	39.5	29.9	6.2	1.6	0.6	0.5
48 h avg	Mean	2435.5	316.8	100.2	84.8	20.8	5.4	1.6	1.4
	SD	805.5	139.1	37.1	34.1	6.7	2.3	0.6	0.6

**FIGURE 3:**
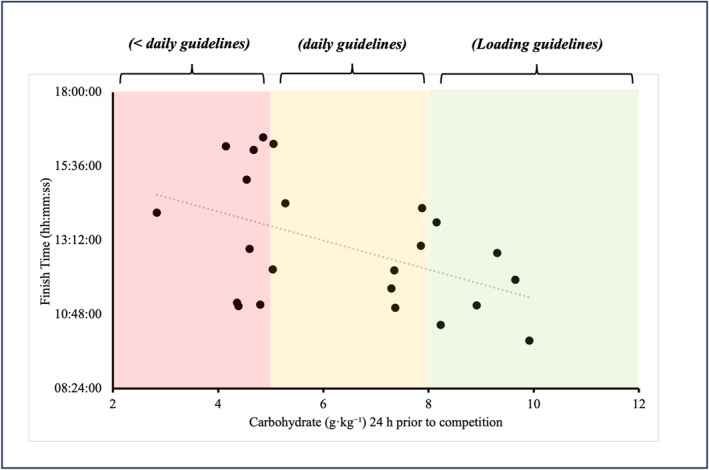
The relationship between carbohydrate intake in the 24 h prior to competition and race finish time. The shaded regions indicate reference ranges for carbohydrate intake based on established nutrition guidelines: red represents intakes below daily endurance recommendations (< 5 g·kg^−1^; Thomas et al. 2016), yellow represents the typical daily target for endurance training (5–7 g·kg^−1^), and green represents evidence‐based carbohydrate loading recommendations (8–12 g·kg^−1^; Burke et al. 2011). The dashed line represents an illustrative linear association.

Relative carbohydrate intake on Day −1 was significantly and negatively correlated with finishing time (*r* = −0.50, 95% CI: −0.76 to −0.11; *p* = 0.035), indicating a moderate inverse association whereby athletes reporting higher carbohydrate intake tended to achieve faster finishing times (Figure 3). The confidence interval suggests that the true effect likely lies between a small and large negative association, reflecting moderate uncertainty given the sample size.

Fibre intake was not associated with finishing time (*r* = −0.18, 95% CI: −0.55 to 0.25; *p* = 0.465), with the confidence interval spanning both negative and positive values, indicating no clear directional relationship. In contrast, fibre intake demonstrated a strong positive association with gastrointestinal (GI) symptom severity (*r* = 0.85, 95% CI: 0.67 to 0.93; *p* < 0.001), suggesting that athletes consuming higher fibre intakes reported greater digestive discomfort during the event (symptom score ≥ 4/10). Carbohydrate intake was not significantly associated with GI symptoms (*r* = −0.13, 95% CI: −0.50 to 0.28; *p* = 0.61), and the wide confidence interval indicates considerable uncertainty regarding the direction and magnitude of this relationship.

Self‐reported behaviours supported these findings. Eighty‐four percent of athletes indicated they deliberately increased carbohydrate intake in the 48 h before competition, which was reflected in the observed increase from Day −2 to Day −1. Reported motivations included professional guidance, the desire to maximise glycogen storage, and ensuring adequate energy availability on race day. Those who did not increase intake commonly cited personal beliefs, previous experiences, or discomfort associated with high carbohydrate feedings. Similarly, 89% reported modifying their hydration strategy, either by preloading electrolytes or increasing fluid intake, whereas others reported no change, having already incorporated electrolyte use into their habitual practise. These behaviours illustrate the heterogeneous nature of preparation and the influence of individual experience, external advice, and performance goals on nutritional decision‐making.

### 3.2 Qualitative

#### 3.2.1 Attitudes: Competer Versus. Completer Mindsets

Athletes' approaches to pre‐race nutrition lay along a continuum from ‘competers’ to ‘completers’. Competers, driven by performance goals such as time or placings, followed structured evidence‐informed fuelling plans and viewed nutrition as integral to success. Completers, aiming mainly to finish or enjoy the event, adopted more flexible intuitive approaches and considered detailed planning less essential.

This mindset influenced how athletes prepared for competition. Competers sought control and precision.

P17 Why would you not want to set yourself up for the most success, or at least for a good experience. I certainly do what I can to at least avoid any big mistakes—it's about doing research but also trial and error.

P16 Prior to a race like this, I'm trying to absolutely flood my muscles with glycogen, as I need that all in there for energy during the race, you know, it's a long day. I also match the carbs with water too, to be able to store it. So, I focus on having that combination and it takes that length of, you know, a day or 48 h to absorb it properly. I often hear people complain about being uncomfortable on the race day as they ate too much the night before, so I have always spread over 2 days. I was told to aim for 8–10g (per kg) of carbs and so I have always aimed for the upper end, but my efficiency and ability to do that has improved over the last couple years.

Amongst competitors, emotions such as stress, excitement, and anticipation shaped how each balanced structure and flexibility. Although carbohydrate loading and meal timing were widely recognised, inconsistent understanding led to variable application.

In contrast, those focussed on completing the race (“completers”) often take a more flexible intuitive approach, shaped by their perception that a rigid plan is less necessary if speed and performance are not primary concerns.

P7 I'm not here to break records or win or anything, so I don't need to be super calculated or do any fancy preparation. I'm not fast, you know, I don't need all that sugar.

P3 I am never going to be the person who counts or calculates these things, I don't need to at my ability, I am just here to get round.

The competer‐completer continuum therefore captures how differing motivations and identities influence the value placed on nutrition during pre‐race preparation. Ultimately, pre‐race preparation is a deeply personal process, shaped by both psychological and nutritional factors that impact performance and experience. This motivational continuum aligned with observed dietary behaviour. Although 84% of athletes reported intentionally increasing carbohydrate intake in the 48 h prior to competition, only 26.1% met carbohydrate loading recommendations (8–12 g·kg^−1^), highlighting a gap between intention and achieved intake.

#### 3.2.2 Subjective Norms: Community, Identity, and External Influences

Athletes' pre‐race routines and nutritional strategies are shaped by a complex mix of personal experience, external influence, and their perceived identity within the triathlon community. Past experiences, social media, coaches, and peers reinforce behaviours that athletes believe are essential for success.

P7 You know I've been and done all the pre‐race dinners and the expedition stuff and whilst it's fun to get engrossed by all the bells and whistles, I'd now rather just spend as much time relaxing and out of that environment until race day, as it can be full on and ultimately, you've got a big race to content with and it's easy to expend a load of nervous energy before your even started. So now I am better at just being chilled and it's much more pleasant for me that way.

P5 Yeah that's a big discussion (carbohydrate loading), because you see different things and there's a lot of different literature on it. But I work with a nutritionist, and she was a specialist and gave me a plan which worked so well, and I have followed that ever since and see that sort of thing work well for others more often too

Additionally, external sources play a key role in shaping nutritional decisions, often guiding athletes towards strategies they associate with high performance.

P4 So classic carb loading for me. Yeah, the week before. I have even read it somewhere that it should be 2 weeks before, but you know everyone's different. I know you should be doing that; you see it everywhere and hear it everywhere and for me that's a lot of pasta, a lot of spinach a lot of broccoli, you know meat, like a lot of chicken.

P16 There's also the pasta party the day before, which I feel like you need to go to, you know it's not an IRONMAN without that

Athlete self‐perception within the sport further influences these choices, with those aiming for peak performance tend to adopt structured approaches, whereas others take a more flexible stance.

P8 I see it a lot in triathlon forums, people saying like ‘oh I am carb loading’ and you know, everyone gets over excited by it, which is fine. But then I just stick to the fact that if I am not going to use it then I don't eat it and because I am slower, I won't need it or use it.

Ultimately, pre‐race preparation is not just a personal process but a socially and culturally influenced practise.

#### 3.2.3 Perceived Behavioural Control: Planning Versus Adaptation

Athletes have a sense of control over key aspects of their race preparation, carefully managing factors such as food access, accommodation, and schedules to foster confidence leading into race day.

P16 Yeah so I chose this place we are staying deliberately, so I can do my own food. I don't want to be tied to a hotel.

P11 Whenever I have a full distance triathlon, I try and stay somewhere with a kitchen so I can cook for myself and control my intake, that's super important.

However, the pre‐race period often presents unpredictable challenges, such as, travel delays, unfamiliar meal options, new environments, and social obligations, which can disrupt even the most rigid plans. Although some athletes embrace flexibility and adapt to these changes, others find the uncertainty stressful.

P14 I won't worry too much, I will eat at restaurants and just choose the cleanest option. It doesn't need to be healthy, just whatever has the least amount of ingredients really and hope that it’s all good.

P4 Travelling is quite a way for me and we will be going by ferry and driving down, so again we can kind of control foods for the most part. Whilst we are there we will be with friends who will want to go out for food and I have said I don't really want to do that but there will be times where we will, so an additional stress there.

Ultimately, successful pre‐race preparation requires a balance between structured planning and the ability to navigate unexpected obstacles.

#### 3.2.4 Utilitarian Drivers: Practical and Comfort‐Based Influences on Pre‐Race Nutrition

In the lead‐up to race day, athletes face a range of external challenges and personal pressures that impact their preparation. Although they work to manage key aspects such as nutrition and logistics, unpredictable elements, such as travel disruptions, unfamiliar food, and cultural differences, can complicate their plans and increase stress.

Additionally, balancing race preparation with the expectations of family, friends, and social obligations can create further strain.

P6 I definitely struggle a lot in saying no socially. So like yeah, my partner is often the one to tell me that I don't need to say yes all the time to everything. That's the same when racing and wanting to do everything the days before and get involved with everything and do what all your friends do.

Gut comfort emerged as a dominant driver of planning and food choice, as a priority in order to promote gut comfort and avoid GI discomfort during competition. This rationale was consistent with the quantitative association between fibre intake and GI symptom severity (*r* = 0.85, *p* < 0.001), despite fibre not being associated with finish time.

P13 I have had runners gut in races before, you know, it's always looming and as a women of a certain age, you have bladder problems too. So especially around bigger and longer events, I tweak what I am doing so I am not loading my gut with too much to deal with.

P21 A lot of what I eat pre‐race is basically in line with how my stomach feels. So you know, you might get the idea of, well, you need to have *x* amount of carbs leading in. But really, if I push my stomach, I can get to the point, like, where I can't even run on the same day if my stomach is no good.

Emotional factors, such as stress and nerves, also influence eating behaviours, with some experiencing a loss of appetite or emotional eating.

P19 I tend to be stressed and yeah, that makes it hard to eat a lot, but I know I have to since the next day is an endeavour, and I will just take it as normally as I can, which would look like boring pasta and staying chilling

Ultimately, athletes must navigate a delicate balance of external disruptions and internal pressures to ensure they are race‐ready.

#### 3.2.5 Behavioural Intentions: Structured Plans and Intuitive Practices

Athletes’ pre‐race plans are very individual, with a variety of different focus points. Although most athletes acknowledge the concept of carbohydrate loading, athletes' opinions on whether this is suitable for them differ. Some plan to flow a rigid plan, which has been carefully designed and tested to increase carbohydrate intake, whereas others (typically completers) do not perceive this approach to be necessary or applicable to them. Some will intuitively increase food intake (across all macronutrients), with the intention to increase carbohydrate intake, but not strictly measure and others will plan to follow normal dietary routines. These planned approaches are driven by an array of factors, from their perceived ability and requirements, performance goals, education, and external influences. Gut comfort is also a driving factor, with many reducing fibre and plan to avoid heavy and rich meals, in attempt to prevent digestive issues. Lastly, in the day prior to racing, many athletes plan to increase their salt and sodium intake through the addition of electrolyte drinks and adding more table salt to foods—this is typically not a calculated process, but rather a subtle increase, with the goal to improve hydration status the following day.

P26 So, I will go for like 10 g (·kg^−1^ BM) of carbohydrate I think it is. Where I just focus on carb loading and lower fibre foods. Lots of rice and easy to digest foods, whilst making sure I stay super hydrated and in the couple days before I refine that even more and eat a really bland and beige diet, with lots of bread, rice and cereal, with a bit of protein.

P20 I will just do what I usually do which is definitely add more carbs and have a bigger meal but not go crazy and have loads because I think that would make you feel awful on the day. I will have bigger portions and reduce vegetables to reduce fibre.

P11 I will stay on my same diet to be honest, carb loading isn't for me, so I stick to normal meals. I just limit gluten to lean down a little more and that's just from experience.

P4 I am not super calculated, i.e. not me, I go off feel. I will always try salt things a lot more in the build‐up though, I consciously do that.

Although many athletes could articulate evidence‐based carbohydrate loading targets (e.g., ‘8–10 g·kg^−1^’), intake data showed high variability, suggesting that knowledge alone did not guarantee implementation, particularly when combined with travel, appetite, and GI constraints, supporting previous observations (Sampson et al. 2024).

#### 3.2.6 Behaviours: From Plans to Practise

Athletes vary widely in their carbohydrate loading strategies, with some following strict intake guidelines whereas others increase portions intuitively or simply make no dietary changes. The different approaches are clearly demonstrated in the large range of intakes in the 24 h prior to the race, which span from 2.83 to 9.92 g·kg^−1^ of carbohydrate (Table 3). Gut comfort is also a key factor, with many consciously reducing fibre, avoiding heavy meals, and making dietary adjustments (e.g., gluten or lactose) to prevent digestive issues (Table 3). Approaches to pre‐race nutrition also differ, with some athletes using calculated numbers‐based plans, whereas others rely on intuition and experience. These differences reflect individual beliefs, with performance‐focused athletes more likely to adopt structured methods, whereas others feel less need for precision.

P23 I had a pretty tight plan, you know, I have done it several times, and it has never failed. I stick to that 10g per kilo (BM), and I do that a couple days before the race. It's quite routine now, but still need to put the steps in like place and you know, have access to the foods I needed, by packing them in my case and then taking snacks with me when we were out and about and that. I stuck to that though and yeah, no stomach issues really so felt fine.

*NB, P23 achieved 9. 92 g·kg* ^*−1*^ *of carbohydrate in the 24 h pre‐race.*

P5 I went out for dinner a couple of times in the build‐up and think I ate something bad, because I was up all night after a meal out being rather unwell

P14 I was on my feet quite a bit, and some periods felt pretty rushed and then you're trying to balance doing all the things you need to with getting the carbs in, so I found myself getting drinks and bars and things and pastries from local shops to keep me going.

*NB, P14 achieved 5.1 g·kg* ^*−1*^ *of carbohydrate in the 24 h pre‐race.*

P1 The build‐up was good. I did the usual pre‐race admin bits and bobs and before you know it, its race day. I ate as usual really, no real plan or carb load, I don't get involved with all of that. So the day before is nothing exciting to be honest. I knew I had a long day ahead the next day so just preparing for that and avoiding anything that would weigh me down up those climbs!

*NB. P1 achieved 2.8 g·kg* ^*−1*^ *of carbohydrate in the 24 h pre‐race.*

**TABLE 3:** Joint display integrating quantitative and qualitative findings interpreted through the ETPB‐X framework.

Quantitative finding	Qualitative insight (illustrative themes/Quotes)	ETPB‐X construct	Integrated interpretation
Only 26.1% (6/23) of athletes met carbohydrate‐loading recommendations (8–12 g·kg^−1^ BM) despite 84% reporting deliberate attempts to increase carbohydrate intake.	Athletes frequently described intentions to increase carbohydrate intake but reported practical barriers such as appetite suppression, logistical constraints, and uncertainty about appropriate foods. Example: ‘I know you should be carb loading… but it's hard to actually get that much in’.	Behavioural intention → behavioural execution	Awareness of carbohydrate‐loading recommendations did not consistently translate into execution. The intention–behaviour gap appears influenced by practical constraints and perceived difficulty in achieving recommended intake levels.
Mean carbohydrate intake increased from 3.9 ± 1.6 g·kg^−1^ (Day −2) to 6.4 ± 2.1 g·kg^−1^ (Day −1) but remained below guideline targets for most athletes.	Athletes described intuitive increases in portion size rather than structured carbohydrate‐loading strategies. Example: ‘I just eat bigger portions the day before rather than calculating anything’.	Attitudes/Behavioural intention	Many athletes recognised the need to increase carbohydrate intake but applied intuitive strategies rather than evidence‐based loading protocols, resulting in suboptimal carbohydrate availability.
Higher carbohydrate intake was associated with faster finishing times (*r* = −0.50, *p* = 0.035).	Athletes with structured fuelling approaches often described a stronger performance orientation and deliberate preparation. Example: ‘Why wouldn't you want to set yourself up for success?’	Attitudes/Athletic identity	Performance‐oriented athletes (‘competers’) appeared more likely to adopt structured fuelling strategies that aligned with carbohydrate‐loading recommendations.
Fibre intake showed a strong positive correlation with GI symptom severity (*r* = 0.85, *p* < 0.001) but no association with finish time.	Athletes frequently prioritised gut comfort, yet many continued to consume foods perceived as ‘healthy’, such as vegetables and whole grains. Example: ‘I eat lots of spinach and broccoli before races’.	Utilitarian drivers	Public health dietary messaging (high fibre intake) may conflict with performance‐specific recommendations for low‐fibre diets prior to endurance competition.
Large inter‐individual variation in carbohydrate intake (2.8–9.9 g·kg^−1^).	Athletes described diverse preparation approaches ranging from structured plans to intuitive or minimal strategies depending on race goals.	Attitudes/Athletic identity	The ‘competer–completer’ continuum influenced the level of nutritional structure adopted in race preparation.
Athletes reported logistical and environmental barriers to following intended nutrition strategies.	Common challenges included travel disruptions, restaurant reliance, and social obligations. Example: ‘It's hard when you're travelling and eating out’.	Perceived behavioural control	Environmental constraints strongly influenced the translation of nutritional intentions into executed behaviour.
Athletes who reported greater control over their food environment (e.g., self‐catering accommodation) described greater adherence to planned nutrition strategies.	Example: ‘I always stay somewhere with a kitchen so I can control my food’.	Perceived behavioural control	Greater logistical control increased the likelihood that athletes could execute intended fuelling plans.
Athletes often modified fibre intake to reduce GI symptoms, although strategies varied.	Athletes described reducing vegetables or choosing simpler foods prior to racing.	Utilitarian drivers	Gut comfort acted as a dominant driver of food choice in the pre‐race period.

#### 3.2.7 Behavioural Determinants

**FIGURE 4:**
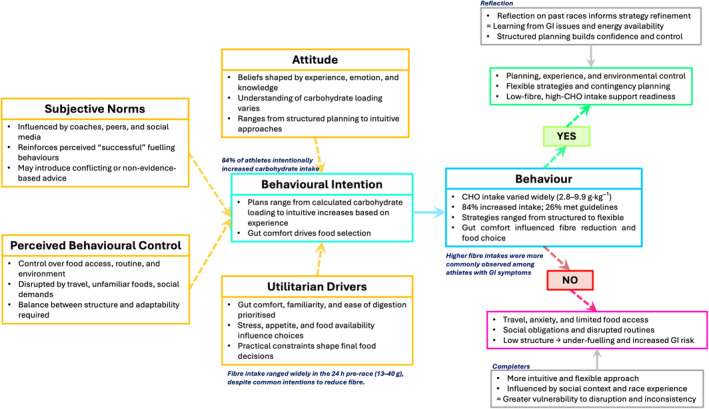
Synthesis of quantitative and qualitative findings mapped onto the Extended Theory of Planned Behaviour–Execution (ETPB‐X) framework (*n* = 27). Yellow boxes represent the original TPB constructs (attitudes, subjective norms, and perceived behavioural control) alongside utilitarian drivers, which collectively shape behavioural intentions (light blue). Behaviour (behavioural execution) is illustrated through green (facilitators) and red (barriers) pathways, reflecting how intentions were enacted under real‐world conditions. Grey boxes represent reflective processes and experiential learning, illustrating how prior experiences inform strategy refinement and behavioural adaptation. Arrows indicate directional relationships between constructs and outcomes, integrating qualitative themes with quantitative findings.

Athletes who reflect on past races use both successes and setbacks to refine their pre‐race strategies, developing rituals and adjusting nutrition plans to address previous issues such as stomach discomfort or energy dips (Figure 4). A well‐structured plan acts as a psychological safety net, reducing stress and fostering confidence in execution. Those who perceive themselves as competitive, whether racing against others or themselves, adopt a more focussed and disciplined approach, planning their nutrition and pre‐race routines to optimise performance. Control over their environment also plays a crucial role, with athletes who choose to self‐cater better able to adhere to their nutrition plans, reducing reliance on unpredictable food sources (Figure 4). Managing the pre‐race atmosphere, whether by limiting social engagements or seeking support from family or friends, further enhances preparations. Structured carb‐loading strategies (high‐carbohydrate, low‐fibre) contribute to improved gut comfort, energy levels, and overall race confidence.

Athletes who view their goal as simply completing the race rather than competing tend to take a more relaxed intuitive approach, often forgoing rigid nutrition plans (Figure 4). Family, social commitments, and engagement in the race atmosphere can further lead to deviations, with those lacking structured or adaptable strategies struggling most. Pre‐race anxiety is frequently experienced by competitive athletes (G. Jones and Hanton 2001) and may disrupt sleep, appetite, and eating behaviours through stress‐related psychophysiological responses (Torres and Nowson 2007; Urwin et al. 2021). Consequently, athletes who feel underprepared or lack effective coping strategies may experience heightened anxiety and greater difficulty adhering to intended nutrition plans in the lead‐up to competition. Limited control over food sources increases vulnerability to issues such as food poisoning or poor meal timing. Logistical hurdles: registration, travel, and last‐minute disruptions can derail nutrition plans, particularly for those who have not accounted for such challenges. However, athletes who build flexibility into their preparations are better equipped to navigate these disruptions and minimise their impact on race‐day performance.

**FIGURE 5:**
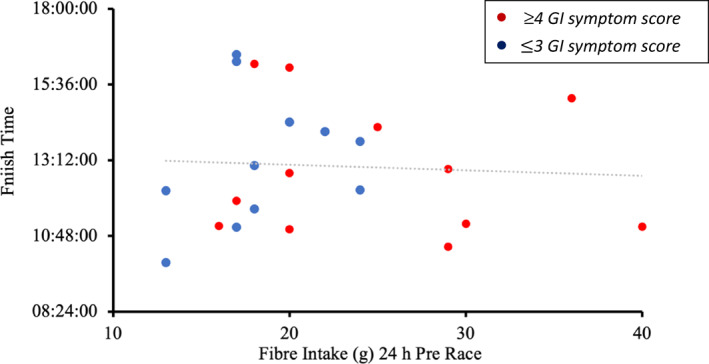
The relationship between fibre intake in the 24h prior to competition and race finish time (hh:mm:ss) (*n* = 23). Red dots denote athletes who reported a GI symptom with the severity of > 4/10, a pre‐established threshold, whereby the symptom is affecting their ability to exercise. Blue dots are for athletes who score symptoms of ≤ 3/10, with the dashed line representing an illustrative linear association.

One common focus for athletes prior to racing was gut comfort and the avoidance of GI symptoms in the build‐up and on race day. Although GI symptoms are commonplace, several strategies are recommended to mitigate risk. Reducing fibre intake is perhaps the most notable strategy and was commonly reported by participants. Despite this, several athletes consumed fibre well above the ∼30 g/day population guideline (WHO/FAO 2003; EFSA Panel on Dietetic Products and Nutrition and Allergies (NDA) 2010), at a time when sports nutrition recommendations emphasise lowering intake, without specifying a set target, to reduce GI risk (Burke et al. 2011; Thomas et al. 2016; IOC (International Olympic Committee) 2018). Data described and integrated with mixed methods interpretation in Table 3. As GI symptoms during the race were measured as part of an additional study, we were able to relate these outcomes to pre‐exercise fibre intake (Figure 5), where higher fibre intakes were associated with more severe GI symptoms during competition.

## 4 Discussion

This mixed‐methods study investigated the pre‐race nutrition practices of female endurance athletes competing at the 2024 IRONMAN World Championships. By combining quantitative intake data with qualitative perspectives and interpreting results through the ETPB‐X, we were able to contextualise the intention‐behaviour gap in pre‐race fuelling. Four key findings emerged: (1) carbohydrate intake in the 48 h pre‐competition was commonly below evidence‐based targets despite widespread awareness of the importance of increasing carbohydrate intake, (2) perceived behavioural control (PBC), particularly logistical autonomy and psychological regulation, was decisive for translating intentions into execution, (3) higher pre‐race fibre intake was strongly associated with GI symptom burden, suggesting a gap between population‐level dietary guidance and sport‐specific recommendations, and (4) reflection and athletic identity substantially shaped intentions and, in turn, behaviours.

### 4.1 Nutritional Practices

Only 26% of athletes (6/23) in this study met carbohydrate loading recommendations of 8–12 g·kg^−1^ BM in the 24–48 h before competition. This aligns with previous work reporting similarly suboptimal intakes amongst endurance athletes. Heikura et al. (2017) observed mean pre‐race intakes of 5.2 ± 1.8 g·kg^−1^ in elite female marathoners, whereas reported 5–7 g·kg^−1^ in IRONMAN triathletes. More recently, Sampson et al. (2023), found mean intakes of 6.5 ± 2.2 g·kg^−1^ in a large international endurance cohort (mixed sex), with only 16% achieving the 9–12 g·kg^−1^ target, despite 36% correctly identifying the guideline. The above intake rates closely match the 6.4 g·kg^−1^ average observed in the present study. Collectively, these findings demonstrate that insufficient carbohydrate loading persists across endurance populations. The performance advantages of meeting carbohydrate loading targets are well established, with greater carbohydrate availability improving glycogen storage, endurance capacity, and time‐to‐exhaustion (Burke et al. 2011). Consistent with this, in the present study, greater carbohydrate intake in the 24 h prior to competition was associated with faster finish times, reinforcing the established performance relevance of carbohydrate adequacy and demonstrating this in the pre‐competition period in female IRONMAN athletes. Importantly this association is unlikely to reflect carbohydrate intake in isolation. Athletes with higher carbohydrate intake may also differ in training status, race experience, pacing strategy, and overall preparation quality. Qualitative findings further suggest that athletes adopting a more competitive orientation (‘competer’ mindset) were more likely to implement structured fuelling strategies, potentially confounding the relationship between carbohydrate intake and finishing time.

Pre‐race fibre intake was strongly associated with greater symptom severity. Several athletes exceeded the ∼25–30 g·day^−1^ population guideline (WHO/FAO 2003; EFSA Panel on Dietetic Products and Nutrition and Allergies (NDA) 2010) at a time when sports nutrition consensus statements explicitly recommend reducing fibre to lower GI risk and to enable higher carbohydrate intakes (Burke et al. 2011; Thomas et al. 2016; IOC (International Olympic Committee) 2018). Mechanistically, high fibre increases stool bulk, fermentation, and colonic gas production, all of which can exacerbate exercise‐induced gut disruption (de Oliveira and Jeukendrup 2014). Despite widespread awareness of carbohydrate loading, fibre management appeared less understood. Athletes frequently relied on familiar or ‘healthy’ foods, such as salads and whole grains, that inadvertently increased pre‐race fibre. This reflects a utilitarian driver: prioritisation of comfort and familiarity over guideline adherence. Stress and anxiety further contributed through gut–brain interactions, which are increasingly recognised as contributors to GI distress in endurance sport (Wilson et al. 2023). Female athletes may be particularly vulnerable, since evidence indicates women report higher rates of pre‐race anxiety and GI complaints compared with men (G. Jones and Hanton 2001; McLean et al. 2011). Practical recommendations should operationalise ‘low fibre’ by providing explicit substitutions, for example, white rice instead of brown rice, or refined cereals instead of whole grains, rather than generic advice. Emerging evidence also suggests that low‐FODMAP interventions may benefit athletes with persistent symptoms, although these should be short‐term, supervised, and balanced against the need for adequate carbohydrate intake (Lis et al. 2018; Wiffin et al. 2019).

To support high carbohydrate availability whilst minimising GI risk in the final 24–48 h before competition, athletes may prioritise low‐fibre high‐carbohydrate foods. Practical examples include:

- Breakfast: white toast with honey or jam, white bagels, rice cereal with low‐fat milk or alternatives
- Main meals: white rice, white pasta, potatoes without skin, low‐fibre wraps with lean protein
- Snacks: rice cakes, low‐fibre cereal bars, pancakes, fruit juice
- Foods commonly reduced: whole grains, legumes, large vegetable portions, high‐fibre fruits

These examples reflect strategies reported by athletes in the present study and align with established endurance nutrition guidance for carbohydrate loading and GI management.

### 4.2 Behavioural Determinants

The TPB provides explanatory value for this noted discrepancy. Attitudes towards carbohydrate loading varied by athlete identity. Performance‐focused athletes (‘competers’) perceived fuelling precision as essential and invested in structured loading protocols, whereas ‘completers’ viewed such practices as unnecessary given their primary goal of finishing the race. This competer/completer distinction has been observed in endurance psychology and highlights the influence of performance orientation on nutrition behaviours (Cerin and Leslie 2008). Subjective norms further shaped behaviours. Coaches, peers, and social media reinforced carbohydrate loading as a norm, but also contributed to inconsistent practices, with athletes citing conflicting or misapplied advice. Previous research confirms that athletes often over‐rely on peer‐shared strategies rather than evidence‐based guidance (Tam et al. 2019; Devlin and Belski 2015; Janiczak et al. 2022; Heikkilä et al. 2018). Decision‐making around food intake in athletes is influenced not only by knowledge or performance goals but also by identity, routine, and environmental constraints, which may explain variability in pre‐race fuelling behaviours observed in the present study (Eck et al. 2021).

Sex‐specific considerations may further influence adherence. Some research suggests that female athletes are less likely than males to meet carbohydrate recommendations, potentially due to differences in energy requirements, sociocultural body‐image pressures, and a higher prevalence of under‐fuelling behaviours (Loucks 2004; Logue et al. 2020, 2021; Fortis et al. 2025; Lodge et al. 2023). This underscores the importance of gender‐specific education for athletes. In this context, sex‐specific education does not imply fundamentally different physiological guidelines, but rather tailored framing, delivery, and application of existing recommendations. This may include addressing misconceptions around carbohydrate intake and body composition, explicitly distinguishing performance‐focused fuelling from everyday health messaging, and acknowledging practical barriers such as appetite suppression, GI tolerance, and anxiety during race preparation. Education that contextualises carbohydrate loading within female athletes lived experiences, rather than assuming guideline awareness translates into execution, may therefore be more effective in supporting adherence in endurance settings.

Athletes' ability to translate intentions into behaviour appears to be strongly determined by PBC. Those who self‐catered or booked accommodation with kitchen facilities described greater control and reported closer adherence to intended plans. Conversely, reliance on restaurants, exposure to unfamiliar cuisines, and meeting social obligations negatively affected adherence. These findings echo TPB predictions that perceived control is the strongest predictor of whether intentions result in action (Ajzen 1991), and they align with prior work highlighting the challenges of nutritional adherence during travel and competition (Burke et al. 2019).

Psychological factors amplified these environmental barriers. Pre‐race anxiety was often associated with a reduced appetite and subjectively disrupted sleep, both of which could disrupt carbohydrate loading efforts. This is consistent with evidence that stress influences appetite‐regulating hormones and gut function (Torres and Nowson 2007), and with prior reports of anxiety‐driven fuelling inconsistencies in endurance athletes (Urwin et al. 2021). Notably, athletes who adopted flexible, adaptive strategies, for example portable carbohydrate sources and contingency meals/snacks, were better able to maintain intake, suggesting that interventions to strengthen PBC should include both logistical preparation, psychological coping strategies, and an element of flexibility. Practical strategies include ‘menu rehearsals’ (trialling restaurant or travel foods in training), ‘gut training’ to improve tolerance of higher carbohydrate intakes, and scenario planning for travel disruptions. These methods directly enhance perceived control and reduce the likelihood that stress or environment derails fuelling plans (Jeukendrup 2017; de Oliveira and Jeukendrup 2014).

Athletes' reflective practise and self‐identity emerged as equally important determinants of pre‐race nutrition as physiological knowledge or logistical control. Many participants described refining strategies across seasons, learning from both successes and setbacks, for example, adjusting carbohydrate timing or avoiding foods linked to previous discomfort. This process of iterative reflection aligns with experiential learning theory, whereby behaviours are refined through cycles of trial, feedback, and adaptation (Kolb 1984). Such reflection is not incidental but central to the development of robust personalised nutrition routines. Athletic identity further shaped behavioural intentions, which appear to exist along a continuum. At one end, athletes with a strong performance orientation (‘competers’), perceived nutrition as integral to success, engaged in in‐depth planning, and expressed firm intentions to adhere to their strategies. At the other end, those motivated more by participation or completion (‘completers’), often framed nutrition as secondary to enjoyment or experience, adopting more flexible intuitive approaches. Many athletes occupied positions between these, shifting along the spectrum depending on race importance, experience, or perceived competitiveness. Both orientations are valued and equally meaningful within endurance sport, reflecting the diverse motivations of athletes. Neither approach is inherently ‘better’ or ‘more correct’; rather, each represents a different way of engaging with the sport, informed by personal goals, identity, and life context. What our findings highlight is not that athletes should shift from one mindset to another, but that small refinements in preparation may support both mindsets. Such refinements can reduce the likelihood of under fuelling, or preventable GI issues, and highlight that fuelling well is not exclusive to competitive athletes. This continuum aligns with sociological perspectives suggesting that self‐perception within a sporting community influences the behaviours athletes deem necessary for success (Brewer and Cornelius 2001). These findings emphasise that nutrition behaviours are not solely biological but also sociocultural and psychological phenomena, shaped equally by identity, belonging, and meaning as by knowledge or ability. Interventions that overlook these factors risk limited impact, even if physiologically sound. Embedding reflective tools, such as structured post‐race debriefs and addressing athletic identity within practitioner support, may enhance behavioural intention and strengthen adherence over time.

### 4.3 Practical Implications

The findings of this study highlight several practical considerations for endurance athletes, coaches, and practitioners working in real‐world competition settings. First, pre‐race fuelling guidance should move beyond generic carbohydrate loading recommendations to explicitly address behavioural execution, recognising that intentions alone do not guarantee effective implementation (Ajzen 1991; Rhodes and de Bruijn 2013). Structured yet flexible plans, developed and trialled in training (Stellingwerff 2012), appear critical for translating nutritional knowledge into practise. Although a strong correlation was observed between pre‐competition carbohydrate intake and finishing time, this relationship should be interpreted cautiously. Athletes who adopted more structured loading strategies, often reflecting a stronger performance orientation or ‘competer’ mindset, may also have been more meticulous across other determinants of performance, including training preparation, pacing strategy, recovery practices, and in‐race fuelling. Additionally, these athletes may simply represent a higher baseline ability level. Consequently, carbohydrate intake likely reflects one component within a broader performance profile rather than an isolated causal determinant of finishing time.

Second, fibre intake warrants greater emphasis within pre‐race education. Simple, pragmatic strategies, such as prioritising familiar lower‐fibre carbohydrate sources and reducing or swapping vegetables, whole‐grains, and other high‐residue foods in the final 24–48 h may meaningfully improve gut comfort whilst supporting higher carbohydrate availability (de Oliveira and Jeukendrup 2014). The wide inter‐individual variability observed across athletes further underscores the importance of individualised approaches that account for performance goals, prior experience, gut tolerance, and contextual constraints, rather than assuming a single optimal strategy. Collectively, these findings support a shift towards integrated, athlete‐centred fuelling frameworks that align nutritional recommendations with behavioural realities in endurance sport.

In addition to nutritional composition, psychological and organisational strategies appear central to successful pre‐race fuelling execution. Athletes reporting higher adherence commonly described behaviours aimed at reducing cognitive load and pre‐race anxiety, including meal planning, repetition of familiar routines, and limiting exposure to socially or logistically demanding environments, strategies shown to support confidence and coping under stress (Fletcher and Sarkar 2012; Michie et al. 2011). Practical strategies to enhance perceived behavioural control may include: (1) pre‐race food environment planning, such as booking self‐catered accommodation or identifying suitable food outlets in advance; (2) ‘practise race’ or simulation days in training, where athletes rehearse carbohydrate‐loading and low‐fibre strategies under realistic logistical conditions; and (3) simple decision‐support tools, such as meal checklists, shopping lists, or portion guides, to reduce cognitive load and improve adherence during travel and taper periods. Conversely, heightened anxiety, disrupted sleep, and competing social obligations were frequently cited as barriers to both appetite and plan adherence. Practical tools such as meal logging, shared planning documents, or checklist‐based fuelling plans may further support behavioural execution by enhancing perceived behavioural control (Martin et al. 2008; Burke et al. 2011). Although such tools do not replace individual experience, they may offer scalable support mechanisms for athletes navigating complex pre‐competition environments.

### 4.4 Limitations

Several limitations should be considered when interpreting the findings of this study. Dietary intake was assessed using weighed food diaries combined with remote food photography. Although this approach enhances ecological validity in free‐living athletes, self‐reported methods remain subject to participant burden, potential under‐ or misreporting, and error in portion‐size estimation (Martin et al. 2008). Image quality and capture conditions may also influence accuracy. To mitigate these limitations, participants received standardised training, recorded all food and fluid intake using both weighing and photographic methods, and were able to provide contextual information. Quantitative data were further triangulated with qualitative interviews, strengthening interpretive validity. GI symptoms were assessed retrospectively immediately post‐race using a validated questionnaire. Although widely used in endurance research, this approach is subject to potential recall bias (Wilson 2017). Additionally, GI symptoms during competition are multifactorial, influenced by factors such as mechanical stress, heat, hydration status, and splanchnic blood flow, which were not directly measured in the present study. The heterogeneity of hormonal status within the cohort, including premenopausal athletes (with and without hormonal contraception) and peri‐/post‐menopausal athletes, represents a further limitation. The present study was not designed to determine the influence of hormonal status on nutrition behaviours, GI symptoms, or performance outcomes, and this remains an important area for future research. The specificity of the study population should also be acknowledged. Participants were recruited from a single, high‐level event (IRONMAN World Championships) and may not be representative of broader endurance populations, particularly those competing at different performance levels or in other event formats. Although this enhances ecological validity within a high‐performance context, it may limit generalisability. Finally, the Extended Theory of Planned Behaviour–Execution (ETPB‐X) framework represents a theoretically informed data‐driven adaptation rather than a formally validated predictive model. Although supported by convergence between qualitative and quantitative findings, it was not tested using structural equation modelling or longitudinal designs. As such, ETPB‐X should be interpreted as a conceptual framework to support understanding of pre‐race nutrition behaviour, rather than a definitive explanatory model.

## 5 Conclusions

This study provides a novel contribution to sports nutrition practise and research by examining the pre‐race nutrition behaviours of female endurance athletes through the ETPB‐X framework, integrating physiological, psychological, and contextual perspectives. Carbohydrate loading was frequently suboptimal, with higher intakes associated with faster finish times, reinforcing its established role in supporting endurance performance. In contrast, higher fibre intake was linked to greater GI discomfort, highlighting the need for clearer athlete education on adapting everyday ‘healthy eating’ habits during race preparation. Behavioural outcomes were influenced by logistical control, emotional regulation, and athletic identity. Athletes identifying as competitive tended to be more structured and proactive in their planning, whereas those with a completer mindset adopted more intuitive and flexible approaches. Collectively, these findings show that pre‐race fuelling behaviour is not solely physiological but is also deeply psychological and sociocultural.

In practise, supporting female endurance athletes requires guidance that aligns nutritional recommendations with the behavioural realities of competition preparation. Enhancing perceived control embedding reflective practise and providing clear performance‐specific nutrition education may help close the gap between intention and execution, translating preparation into performance. This insight offers a critical bridge between laboratory evidence and applied performance by recognising the psychosocial and contextual barriers that shape nutritional behaviour.

Building on these findings, subsequent work within the same athlete cohort, with the addition of a professional subgroup, will examine the execution of fuelling strategies during competition, including in‐race carbohydrate intake, GI responses, and behavioural adaptations, to provide a more comprehensive understanding of nutrition behaviour across the endurance performance continuum.

## Conflicts of Interest

The authors declare no conflicts of interest.

## Supporting information


Supporting Information S1

## Data Availability

The data that support the findings of this study are available from the corresponding author upon reasonable request.
