# Supplementary material for: From Intention to Execution: Pre‐Race Nutrition Behaviours, Influences, and Performance Outcomes in Female Endurance Athletes at the IRONMAN World Championships
Source: Eur J Sport Sci. 2026 Jun 11;26(7):e70193. doi: 10.1002/ejsc.70193 (PMC13256053; doi:10.1002/ejsc.70193)
Supplement: Supplementary file 1 — Supporting Information S1 [file EJSC-26-e70193-s001.docx]

| **TPB Construct / Domain** | **Core Questions** | **Example Probing Prompts** | **Purpose / Rationale** |
| --- | --- | --- | --- |
| Opening / Context Setting | Can you tell me a bit about yourself as an and your background in endurance sport?  How do you normally approach nutrition in the lead-up to races, what have you done previously?  What does your typical race-week routine look like? | Has this changed much over the years?  Who helps you with this, if anyone?  How are you feeling for the upcoming race? | To establish rapport, understand experience level, and situate later responses. |
| Attitudes | What are your overall thoughts and focuses on nutrition prior to a race?  How important do you think nutrition is for your race performance or experience?  What does ‘getting it right’ or success look like to you? | How confident do you feel that your nutrition choices make a difference?  Why do you think that?  Have you always felt this way?  Do you have any concerns? | To explore beliefs, perceived importance, and emotional tone around nutrition. |
| Subjective Norms | Is there anything/anyone you look towards for guidance?  Do you get advice from coaches, peers, or online sources?  How do you decide which advice to follow? | Are there common practices you see in your training group or the wider triathlon community?  Do you ever feel pressure to prepare in a certain way?  Why do you trust that?  Where did you learn that? Where did you hear this? | To identify cultural, social, and informational influences shaping norms and expectations. |
| Perceived Behavioural Control (PBC) | How much control do you feel you have over what you eat in the days before a race? Do you think you will stick to you intended plan?  What makes it easier or harder to follow your intended plan?  What challenges do you usually face or do you think you may face when trying to stick to your plan? | How are yo planning to manage travel, accommodation, food options?  How do/will you manage when something unexpected happens, like delays or stress? | To understand the role of environment, logistics, and psychological control in shaping behaviour. |
| Utilitarian Drivers | What practical things guide your food choices before racing?  Do you ever choose foods because they’re easy, familiar, or comforting?  How much does gut comfort or nerves affect what you eat? | Do you have any ‘go-to’ meals or rituals that help you feel ready?  Do you make trade-offs between what’s comfortable and what you think might help performance? | To explore comfort, practicality, and emotion-based influences on food choice. |
| Behavioural Intentions (Pre-Race Planning) | Can you walk me through how you plan your nutrition in the days before a race?  Do you have a set plan for what you’ll eat or is it more flexible? When do you start thinking about your race-week meals?  Have you practiced any of the strategies? | Do you aim for specific goals like carbohydrate loading or just eating more generally?  Will you make deliberate changes to your food (fibre, macros, fluids, or portion sizes)? | To assess how intentions are formed, structured, and aligned with performance goals. |
| Reflection and Experience | Thinking back to previous races, how have your pre-race nutrition strategies changed?  What have you learned from times things went well or didn’t go as planned?  Do you think experience has made you more confident in your approach? | Can you recall a key learning moment that changed your preparation? | To explore experiential learning and how reflection shapes current beliefs and actions. |
| Closing | Is there anything else about your race-week nutrition or preparation that we haven’t covered but you think is important? | Anything you wish more athletes or coaches understood about this part of race prep? | To capture final reflections and ensure participant voice completeness. |

**Supplementary material- . Semi-structured interview guide mapped to the Extended Theory of Planned Behaviour–Execution (ETPB-X) framework.**
The table outlines the key domains explored during pre-race interviews, including attitudes, subjective norms, perceived behavioural control (PBC), utilitarian drivers, and behavioural intentions, alongside opening, reflection, and closing components. For each domain, core questions and example probing prompts are provided to facilitate in-depth exploration of athletes’ nutrition beliefs, planning processes, and anticipated behaviours in the lead-up to competition. The guide was designed to capture both cognitive (e.g., beliefs, knowledge, perceived importance) and contextual (e.g., logistics, environment, social influences) determinants of pre-race nutrition, supporting alignment with the ETPB-X framework and enabling insight into the formation and anticipated execution of nutrition-related behaviours.
